# Ultrasound-Guided Percutaneous Achilles Tendon Repair for Acute Achilles Tendon Rupture: Modified Percutaneous Achilles Repair System Procedure for Achilles Tendon Repair Without a Jig

**DOI:** 10.7759/cureus.77730

**Published:** 2025-01-20

**Authors:** Takahide Sasaki, Kazuyoshi Minamino, Yukihiro Nakagawa, Hiroshi Yamada

**Affiliations:** 1 Department of Orthopaedic Surgery, Wakayama Medical University Kihoku Hospital, Wakayama, JPN; 2 Department of Orthopaedic Surgery, Wakayama Medical University, Wakayama, JPN

**Keywords:** achilles tendon rupture, minimally invasive surgery, percutaneous achilles tendon repair, sural nerve injury, ultrasound-guided surgery

## Abstract

Surgical methods for treating Achilles tendon ruptures include open and minimally invasive surgery (MIS). MIS offers notable advantages, including reduced rates of infection and wound necrosis; however, it also introduces the risk of sural nerve injury. Intraoperative ultrasonography can mitigate this risk by providing real-time visualization of the sural nerve and Achilles tendon, improving clinical outcomes. This article introduces a novel ultrasound-guided suturing technique for Achilles tendon repair using a percutaneous Achilles repair system (PARS). This technique does not require the use of a jig, which is conventionally used in the standard PARS procedure to ensure accurate and controlled percutaneous suture placement in the Achilles tendon, making it less invasive than conventional methods. Additionally, it allows for more precise intraoperative visualization of the sural nerve using ultrasonography, enabling safer Achilles tendon repair.

## Introduction

Achilles tendon rupture is a relatively common injury with a reported annual incidence rate of 12.8-13.9 per 100,000 individuals in Japan [1]. Available treatment methods include both conservative and surgical approaches. Traditionally, conservative management has been associated with a higher re-rupture rate than surgery [2]; however, recent research has shown that combining conservative treatment with functional rehabilitation, including early range-of-motion exercises, can achieve re-rupture rates comparable to surgical outcomes [3]. Therefore, the optimal management approach for Achilles tendon ruptures is open for debate. However, surgical treatment is increasingly preferred for Achilles tendon ruptures in Japan [1]. Furthermore, surgical intervention is commonly selected for high-performance athletes desiring early resumption of training activities [4].

Surgical management of Achilles tendon ruptures includes open surgery and minimally invasive surgery (MIS) [5]. MIS for Achilles tendon rupture was first reported by Ma and Griffith in 1977, describing a percutaneous suturing method [6]. MIS comprises both percutaneous techniques and minimally invasive methods utilizing guiding instruments [5]. Compared with open repair, MIS reduces the risk of infection, wound necrosis, scarring, and adhesions [5,7]; however, it may pose a greater risk of sural nerve injury and insufficient tendon repair [5,8]. Intraoperative ultrasound has addressed these challenges, yielding promising clinical outcomes [8,9]. Ultrasound enables real-time visualization of the sural nerve and Achilles tendon, which helps to avoid nerve damage and improves the accuracy of tendon repair [8,9].

Specialized devices have been developed to enable strong and minimally invasive repair of Achilles tendon ruptures. The Percutaneous Achilles Repair System (PARS) (Arthrex, Inc., Naples, Florida, United States) represents a minimally invasive approach for Achilles tendon repair. This technique employs a specialized jig to achieve precise percutaneous suture placement, enabling strong tendon repair with locking sutures while minimizing the size of the skin incision [10,11]. The standard PARS technique for repairing Achilles tendon ruptures requires the insertion of a jig at the rupture site to guide the needle and suture percutaneously through the tendon, followed by the removal of the jig to retrieve the suture from the site [11]. Ultrasound-guided PARS for Achilles tendon repair has been reported. Cross et al. demonstrated that intraoperative ultrasound in the PARS technique mitigates the risks of MIS for Achilles tendon rupture, including sural nerve injury [12]. Despite its advantages, this technique is associated with certain limitations. First, a 3-cm transverse incision is required to insert the PARS jig [11]. Additionally, the insertion and positioning of the PARS jig at the designated site for needle placement in Achilles tendon repair can result in interference with the ultrasound probe. This interference impedes proper skin contact with the probe, complicating the accurate visualization of the sural nerve. In response to these limitations, we developed a modified method. Under ultrasound guidance, the needle and suture were introduced percutaneously through the Achilles tendon without a jig, and an arthroscopic retriever was used to retrieve the suture to facilitate tendon repair.

This report presents a novel, jig-free, ultrasound-guided PARS technique for Achilles tendon ruptures that provides a less invasive option with improved intraoperative visualization of the sural nerve and enhanced suture placement accuracy.

## Technical report

Preoperative planning

The location of the Achilles tendon rupture is evaluated preoperatively using ultrasound or magnetic resonance imaging. An adequate length of the distal stump of the Achilles tendon is essential to accommodate five sutures, meeting the criteria for this technique. In cases with an insufficient distal stump, alternative techniques, such as the Achilles MidSubstance Speed Bridge repair method, should be considered [13].

Patient setup

**Figure 1:**
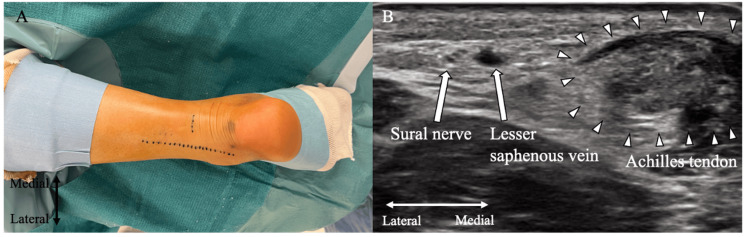
Ultrasound-guided preoperative marking of the sural nerve A: Sural nerve pathway; B: Ultrasound visualization of the sural nerve Image source: Authors

The procedure is performed under a sciatic and saphenous nerve block, lumbar spinal anesthesia, or general anesthesia with the patient positioned prone. Tourniquets are not used. Preoperatively, the contralateral Achilles tendon resting angle (ATRA) is assessed and used as a reference for determining the ATRA after tendon repair. ATRA reflects the position of the ankle joint, measured with the patient positioned prone, the knee flexed at 90°, and the ankle joint fully relaxed [14]. The distal portion of the surgical limb, starting from the knee joint, is disinfected and prepared with sterile draping. The ankle is placed on a cloth or spacer to allow unrestricted dorsiflexion and plantar flexion. The course of the sural nerve is identified and marked preoperatively under ultrasonographic guidance (Figure 1A). On ultrasound, the sural nerve appears as a structure measuring approximately 1-2 mm in diameter, positioned lateral to the Achilles tendon and adjacent to the small saphenous vein. It exhibits typical peripheral nerve sonographic characteristics, including an oval shape and a "honeycomb" appearance in transverse view, formed by small hypoechoic dots interspersed within hyperechoic regions [15]. No acoustic shadowing is observed (Figure 1B).

Suture passage for the proximal stump of the Achilles tendon

**Figure 2:**
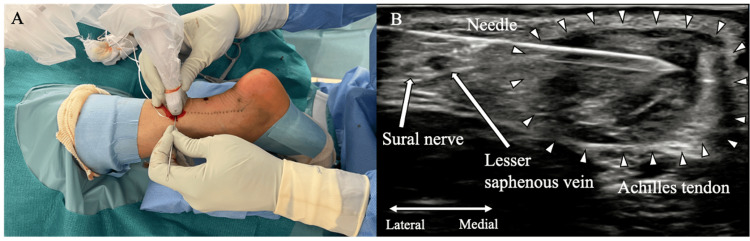
Percutaneous needle insertion into the proximal Achilles tendon stump performed under ultrasonographic guidance to avoid sural nerve injury A: External view of the procedure; B: Ultrasound visualization of the needle passage Image source: Authors

**Figure 3:**
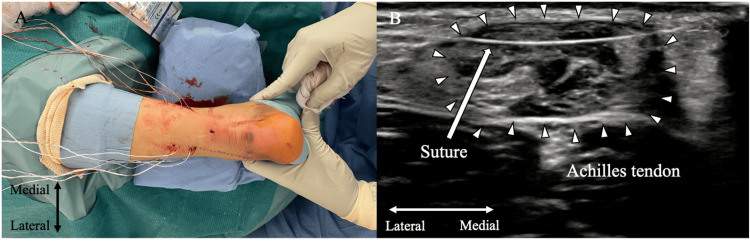
Sutures introduced into the proximal Achilles tendon stump via a needle loop under ultrasound guidance A: External view after suture passage; B: Ultrasound visualization after suture passage Image source: Authors

The PARS kit, which includes a nitinol-looped needle, 1.3-mm suture tape, and fiber link suture with a loop for suture relay, is utilized without the PARS jig in this technique. Ultrasound guidance provides a long-axis view of the proximal Achilles tendon to determine the precise needle insertion points. Approximately 3 cm from the proximal end, needle #1 is inserted laterally and medially to guide the white suture tape #1. A short-axis view of the tendon and sural nerve is used during needle insertion to maintain the central tendon positioning and avoid sural nerve injury (Figure 2). Subsequent needles are placed at intervals of at least 5 mm with the #2 blue/white suture tape, #3 and #4 green/white fiber links, and #5 black/white suture tape threaded through the needle loops and advanced upon needle withdrawal (Figure 3). In acute Achilles tendon ruptures, the frayed appearance of the distal stump may compromise suture strength; therefore, a robust area near the rupture site is chosen for #5 needle placement under ultrasound guidance. For green/white fiber links #3 and #4, each looped end is positioned on the opposing tendon side, consistent with the original PARS technique. After completing the suture passage, the long- and short-axis ultrasound views confirm the central tendon placement of all sutures and avoidance of the sural nerve. Each suture tail is trimmed to an equal length.

Locking suture technique for the proximal stump of the Achilles tendon

**Figure 4:**
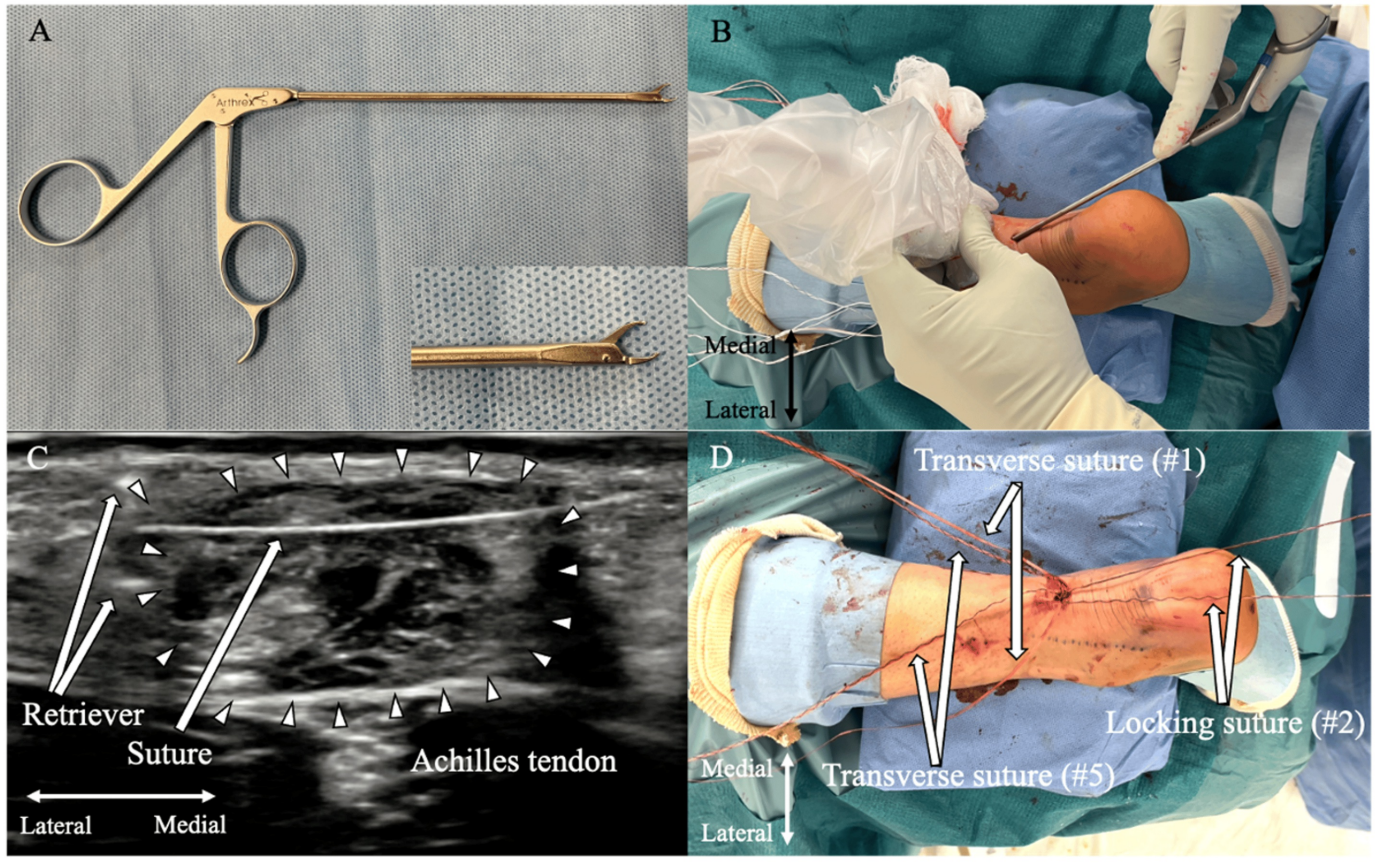
Suture retrieval from the proximal Achilles tendon stump under ultrasound guidance, followed by locking suture placement A: Suture retriever tool; B: External view during ultrasound-guided suture retrieval; C: Ultrasound visualization of suture retrieval; D: Final view after retrieval and locking suture placement Image source: Authors

A 5-mm longitudinal incision is made above the Achilles tendon rupture site, and the paratenon is opened along the same axis to expose the rupture site. A dual-ended elevator raspatory (Tanaka Medical Instruments Co. Ltd., Kita City, Tokyo, Japan) is inserted from the rupture site into the deep paratenon with circumferential dissection of the proximal stump guided by a short-axis ultrasound view. An arthroscopic suture retriever (Arthrex Inc.) (Figure 4A) is inserted through the incision into the rupture site (Figure 4B). Under ultrasound guidance, each suture tape and fiber link on the medial and lateral sides of the Achilles tendon are individually retrieved from the deep paratenon and removed through the incision. During this process, ultrasonography confirms the positions of the Achilles tendon, sural nerve, and sutures, with particular care taken to avoid the sural nerve when retrieving the lateral sutures (Figure 4C).

The sutures are organized proximally to distally on each side in the following order: #1, white suture tape; #2, blue/white suture tape; #3, green/white fiber link; #4, green/white fiber link; and #5, black/white suture tape. The #2 blue/white suture tape is wrapped twice around the #3 and #4 green/white fiber link and then threaded through their looped ends. Pulling the non-looped ends of each green/white fiber link (#3 and #4) guides the #2 blue/white suture tape across the tendon, securing the locking suture. This configuration results in two transverse sutures (#1 and #5) and one locking suture (#2) (Figure 4D).

Suture passage and locking suture of the distal stump of the Achilles tendon

**Figure 5:**
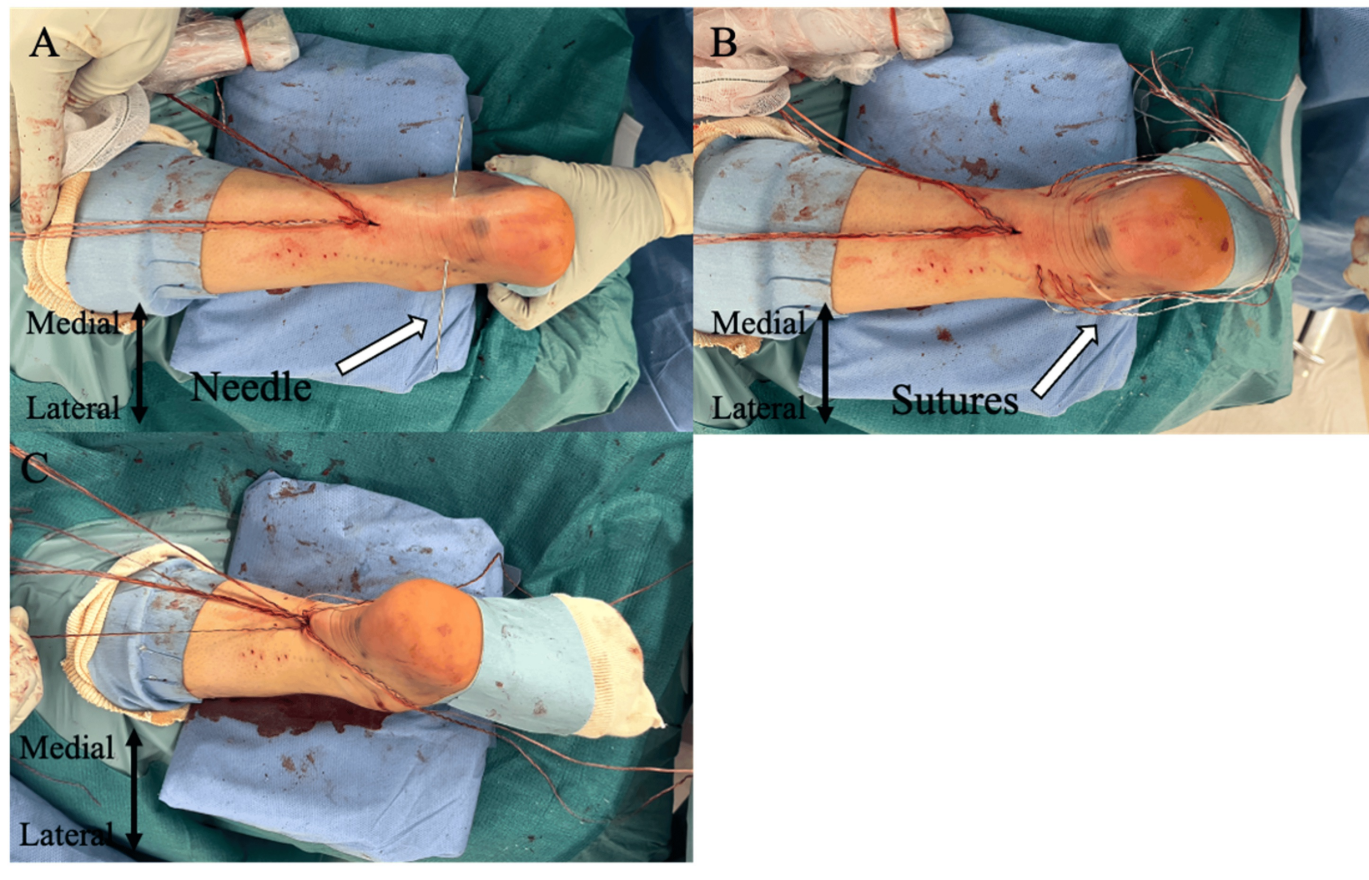
Suture passage and locking suture at the distal Achilles tendon stump under ultrasonographic guidance, replicating the proximal stump technique A: Needle insertion at the distal stump; B: Completion of suture passage; C: Completion of suture retrieval and locking suture Image source: Authors

Following the same principles as those for the proximal stump, suture passage and locking of the distal stump are performed under ultrasound guidance. The needle is inserted from the lateral side during the suture passage for the distal stump. As the sural nerve diverges from the Achilles tendon on the distal side, the risk of nerve injury is significantly reduced compared to that on the proximal side. Sutures are retrieved through the same skin incision used for the proximal stump, and suture locking is completed (Figure 5).

Suture tying of the Achilles tendon

**Figure 6:**
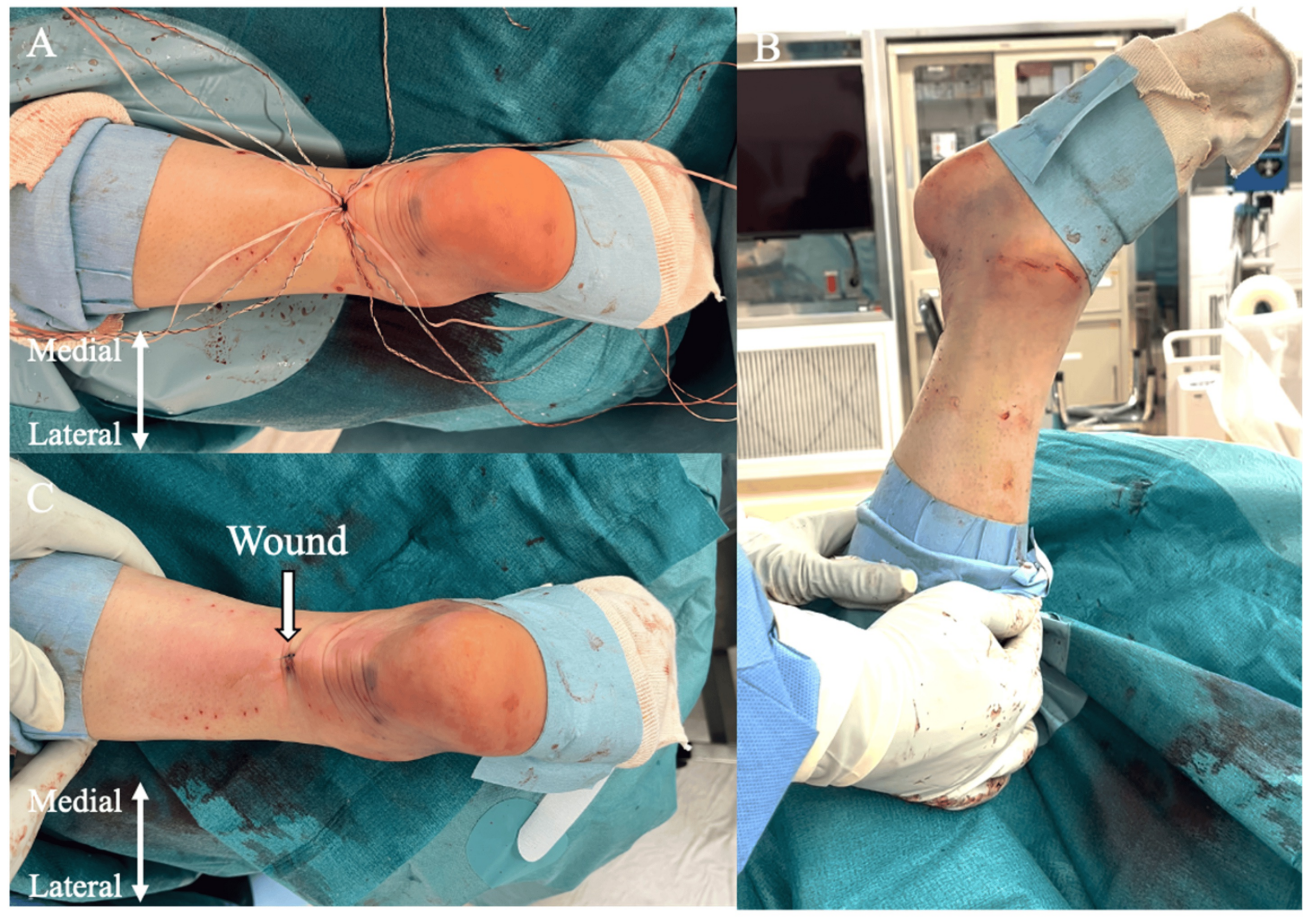
Suture tying of the Achilles tendon with the ankle held in maximum plantar flexion A: Final appearance after suture tying; B: Post-suturing view of the Achilles tendon; C: Surgical wound appearance Image source: Authors

Three suture tapes are extended from the proximal and distal stumps of the Achilles tendon. Each tape is pulled 10 times to eliminate slack. The sutures are tied in sequence as follows: #5 black/white suture tape, #2 blue/white suture tape, and #1 white suture tape (Figure 6A). Following a previous study [10], the sutures are tied under maximum tension with the ankle in plantarflexion to prevent Achilles tendon elongation. The postoperative ATRA is confirmed to exceed the plantarflexed position of the contralateral side (Figure 6B). For the #5 black/white suture tape and #1 white suture tape, the first tied side functions as a stay stitch, and the opposite side regulates tendon tension and finalizes the repair. The #2 blue/white suture tape locks the tendon ends, preventing sliding and enabling precise tension adjustment during suture tying. Each suture is secured using four surgeon’s knots. Ultrasound imaging is employed post procedure to verify proper contact of the Achilles tendon ends and confirm that all knots are embedded within the subcutaneous tissue.

Closure and casting

The skin is closed using superficial no. 4-0 nylon horizontal mattress sutures (Figure 6C). The wound is covered with dry sterile dressings, and a short-legged, well-padded fiberglass cast is applied in the ankle position achieved after surgery.

Postoperative care

Postoperative early weightbearing combined with early ankle motion exercises leads to superior and faster functional recovery after surgical Achilles tendon repair [16]. Early functional rehabilitation is applied following surgery. The patient remains non-weight-bearing during the first postoperative week with a short-leg cast. After one week, the cast is removed, and a walking boot with a heel wedge (Ankle-Foot Orthosis for Achilles Tendon; ADVANFIT Inc., Kumamoto, Japan) is applied. Ankle range-of-motion exercises are initiated at the time of cast removal. Weight-bearing is permitted as tolerated by the walking boot, which contains four heel wedges removed weekly. The boot is applied for five weeks.

Preventing Achilles tendon elongation is vital because elongation may result in reduced calf muscle volume and lasting deficits in plantar flexion strength [17]. To monitor elongation, the patient is placed in the prone position with the ankle hanging naturally, ensuring that the operated ankle does not dorsiflex beyond the non-operated side. Bilateral heel raises are introduced at six weeks postoperatively, followed by single-leg heel raises at eight weeks. Once single-leg heel raises are achieved, jogging can begin between 10 and 12 weeks postoperatively. Jumping and stepping exercises are initiated at four months, with a return to sports typically occurring within five months.

## Discussion

This report introduces a modified PARS technique that omits the use of a jig for the surgical management of acute Achilles tendon rupture. Surgical options for this condition include open repair and MIS such as percutaneous repair [5]. While the ideal method has not been established, meta-analyses have provided comparative insights. Yang et al. observed that percutaneous repair reduced the incidence of deep infections but increased the risk of sural nerve injury compared with open repair [18]. Similarly, Gatz et al. reported that MIS was associated with lower rates of wound necrosis, superficial and deep infections, and scar adhesions, but it demonstrated higher incidences of sural nerve injury and palpable knots [5]. Sural nerve injury remains a major concern in MIS, particularly during percutaneous needle insertion in the proximal Achilles tendon [5]. To address this complication, intraoperative ultrasound guidance was utilized in this study to minimize the risk of sural nerve damage. 

Intraoperative ultrasound has been demonstrated to effectively mitigate the risk of sural nerve injury in MIS for acute Achilles tendon ruptures. Giannetti et al. documented no cases of sural nerve injury in their cohort of 40 patients who underwent percutaneous repair with intraoperative ultrasound guidance [19]. Similarly, a prospective randomized study by Samy compared outcomes between patients undergoing percutaneous repair with intraoperative ultrasound (n = 47) and those without it (n = 44) [8]. The group without ultrasound reported two cases of re-rupture and two cases of sural nerve injury, while no complications were observed in the ultrasound-assisted group. These findings highlight the critical role of intraoperative ultrasound in minimizing the risk of nerve damage during MIS for Achilles tendon repair. In this technical report, ultrasound guidance was utilized to avoid the potential sural nerve injury associated with blind needle insertion and suture retrieval. Therefore, the integration of ultrasound into MIS protocols for Achilles tendon repair is imperative for optimizing patient safety and reducing complications.

This technique represents a modification of the PARS method, which employs a specialized device for minimally invasive and robust Achilles tendon repair. PARS has demonstrated favorable clinical outcomes in treating acute Achilles tendon ruptures. Hsu et al. evaluated 101 cases repaired with PARS and reported that 98% of patients returned to baseline physical activities within five months [10]. Minor complications, including three cases of superficial wound dehiscence and two cases of superficial foreign-body reaction to FiberWire, were observed, with no incidences of sural nerve injury or re-rupture [10]. The PARS method uses a jig inserted beneath the paratenon to retrieve sutures percutaneously, effectively reducing the risk of incorporating the sural nerve into the sutures when the jig is properly positioned. However, percutaneous needle insertion into the Achilles tendon poses a potential risk of direct sural nerve injury. To mitigate this risk, Cross et al. introduced ultrasound-guided PARS, which significantly reduces the risk of sural nerve damage compared to conventional PARS [12]. Nonetheless, according to the experience of the authors of the present report, the insertion of the jig creates interference with the ultrasound probe, hindering adequate contact with the skin. This interference complicates the visualization of the sural nerve and the needle insertion into the Achilles tendon under ultrasound guidance. By omitting the jig, the procedure described in this report simplifies these technical challenges.

This technique offers distinct advantages, including enhanced visualization of the sural nerve via intraoperative ultrasound, reduced incision size for PARS jig placement, and the facilitation of robust locking sutures using the PARS system. First, it requires advanced proficiency in ultrasound-guided interventions. The accurate placement of needles into the Achilles tendon and the retrieval of sutures under ultrasound guidance are essential steps that demand both expertise and experience. Surgeons who lack sufficient familiarity with these techniques may encounter reduced surgical success rates. Second, the locking suture strength achieved by this method may be inferior to that of the standard PARS technique with a jig. In the standard PARS technique, the #3 green/white fiber link and #4 green/white fiber link are placed diagonally across the Achilles tendon, whereas in this technique, these fiber links are aligned parallel to the tendon. Biomechanical studies have shown that the locking sutures used in the standard PARS method provide greater tensile strength than Krackow sutures [20]. Although the suture strength of this technique has not been validated, it is expected to achieve sufficient strength, as it employs the same locking suture mechanism as the standard PARS method. Third, the retrieval of sutures using an arthroscopic retriever remains a technically demanding aspect of the procedure. Innovations in both surgical techniques and ultrasound-guided instrumentation are necessary to enhance the safety and reliability of minimally invasive approaches for Achilles tendon repair. Finally, as the advantages of this method are currently based on theoretical assumptions, further studies are necessary to validate its efficacy.

## Conclusions

This report describes a novel ultrasound-guided percutaneous Achilles tendon repair technique that offers a less invasive approach than conventional ultrasound-guided PARS methods and significantly reduces the risk of intraoperative sural nerve injury. This technique enhances the real-time ultrasound visualization of the sural nerve by eliminating the need for a jig. It improves the precision of needle insertion into the Achilles tendon, thereby ensuring safer and more reliable suturing. Further studies are required to validate the long-term efficacy and safety of this treatment.
